# Healthcare worker perspectives on barriers to integrating mental health services into human immunodeficiency virus care in Windhoek, Namibia: A convergent parallel mixed-methods design

**DOI:** 10.4102/jphia.v17i1.1798

**Published:** 2026-06-18

**Authors:** Ndeshiteelela K. Conteh, Ozayr Mahomed

**Affiliations:** 1 School of Health Sciences, Faculty of Nursing and Public Health, University of KwaZulu-Natal, Durban, South Africa; 2 Dasman, Diabetes Institute, Kuwait City, Kuwait

**Keywords:** mental health, mental health integration, barriers to care, Namibia, people living with HIV

## Abstract

**Background:**

Mental illnesses are more prevalent in people living with HIV (PLHIV) than in the general population, highlighting the need to integrate mental health services into primary healthcare (PHC). Health workers are key players in integrating these services.

**Aim:**

This study assessed healthcare workers’ perceptions, opinions, and experiences on barriers to integrating mental health services into antiretroviral therapy (ART) services.

**Setting:**

The study was conducted in public health facilities in Windhoek, Namibia.

**Methods:**

A descriptive mixed-methods study using a convergent parallel design was conducted among 44 healthcare workers (*n* = 44), including 10 in-depth interviews. Data were collected through questionnaires and in-depth interviews. Quantitative data were analysed using STATA version 15, while qualitative data were analysed using NVivo 12.

**Results:**

The study identified several barriers, including inadequate facility space, limited knowledge and skills for managing mental illness, insufficient in-service training, a lack of guidelines and standard operating procedures, and staff shortages.

**Conclusion:**

Significant structural and workforce-related barriers hinder effective mental health screening, diagnosis, and treatment for PLHIV in PHC settings. Addressing these challenges requires facility restructuring, workforce training, task shifting, and strong political commitment to strengthen healthcare infrastructure.

**Contribution:**

The study provides valuable insights into healthcare workers’ experiences and contributes much-needed regional evidence on barriers to integrating mental health services into HIV care in Namibia.

## Introduction

People living with human immunodeficiency virus (PLHIV) experience higher rates of mental health issues compared to the general population.^1^ While depression and anxiety affect approximately 4% – 7% and 3% – 5% of the general population, respectively, prevalence among PLHIV is substantially higher.^2^ Across Africa, studies report considerable variability: In Cape Coast, Ghana, 28.6% of PLHIV had depression, 40.8% anxiety, and 10.6% stress^3^; in Côte d’Ivoire and Senegal, 17.9% of older PLHIV experienced depression^4^; in Abuja, Nigeria, 28.2% had depression, 2.8% suicidal ideation, and 7.8% alcohol use disorder^5^; and in Cape Town, South Africa, 56% had at least one mental disorder at baseline, with 48% affected after 6 months.^6^ The observed differences in prevalence across settings likely reflect variations in study design, measurement instruments, population characteristics, sociocultural factors, and differences in health system capacity to detect and manage mental health conditions. Recognising this disparity, the World Health Organization (WHO) and Joint United Nations Programme on HIV/AIDS (UNAIDS) have emphasised integrating mental health into HIV care, providing targets and tools to facilitate service integration.^7,8^ In response to the WHO recommendations, various mental health integration models at the primary healthcare level have been documented. Integration models include increasing mental health screening and treatment in antiretroviral therapy (ART) clinics, incorporating HIV care into mental health services, and establishing sub-speciality clinics for PLHIV with mental illness. In Namibia, mental health and HIV services remain largely separate. While HIV care is integrated at the primary health clinic (PHC) level, mental health services are centralised in two psychiatric hospitals. In 2020, only two facilities offered outpatient mental health services,^9^ despite the country having 309 health centres, 34 district hospitals, and four intermediate/referral hospitals.^10^

Evidence on healthcare workers’ (HCWs) perceptions of integration is limited,^11^ though their perspectives are critical for delivering quality care and supporting integration. Focusing on healthcare workers’ behaviours provides opportunities to improve patient outcomes and advance the science of behaviour change, as these behaviours are influenced by professional training, clinical setting, and patient needs.^12^ Notably, integrating services is complex and requires trained human resources and organised workflows^10^ to succeed, and significant care gaps persist between recommended and actual patient care.^13^ Therefore, identifying barriers to mental health service integration from the healthcare worker perspective is key to successful integration.

This study aimed to assess healthcare workers’ perceptions, opinions, and experiences on barriers to integrating mental health services into ART services in Namibia.

## Research methods and design

### Study setting

This study was conducted in Windhoek, Namibia’s capital. In 2023, Windhoek’s population was 494 605.^14^ Windhoek has 12 public health facilities that provide ART services to PLHIV and one national psychiatric hospital.

### Study design

A descriptive mixed-methods study with a convergent parallel design was conducted to explore healthcare providers’ opinions and experiences regarding mental health services for PLHIV at ART clinics and the National Psychiatry Hospital in Windhoek, Namibia. This design enabled researchers to gather deeper insights, triangulate, and corroborate on challenges and gaps in mental health service delivery by integrating qualitative and quantitative findings.^15^

### Population and sampling methods

Ten out of 12 public health facilities were selected based on the number of patients on ART. Healthcare workers providing ART care to PLHIV at ART sites and psychiatric outpatient departments were eligible to participate in the study. The study employed a non-probability snowball sampling method. Snowball sampling was used to recruit participants because the study targeted a specific group of healthcare workers experienced in providing ART and mental health services, which is difficult to identify through random sampling. A total of 44 (*N* = 44) healthcare workers participated in the survey, and 10 in-depth interviews (IDIs) were conducted. Of the 44 participants, 35 were health workers at the ART site, and 9 were from the mental health site.

The required sample size for the study was determined by considering findings from prior research, guidance from research books, and the specific phenomena under investigation. Literature suggests an appropriate sample size for a phenomenological study is 3 to 25 participants.^16^ We followed the data saturation principle, as saturation is the most essential element of data adequacy in qualitative research.^17^ This principle ensures that the data’s quantity (detailed description) and quality (comprehensive description) are sufficient. Questionnaires were self-administered by healthcare workers, while in-depth interviews were conducted in person with study participants.

### Data collection and procedure

Data collection tools were developed in alignment with the study objectives and informed by a review of relevant literature on health workers’ perspectives regarding mental health and HIV service integration. The data collection tools gathered demographic data and items addressing key domains, including knowledge, attitudes, perceived barriers, and experiences related to integrating mental health services into HIV care. The data collection tools were reviewed for clarity and relevance by public health and HIV programme implementation experts. Subsequently, the questionnaire and interview guide were piloted with ART clinic healthcare workers to ensure clarity, and feedback was incorporated into the final versions of the tools. Data were collected in September 2023 and healthcare workers in urban ART clinics in Namibia, providing a snapshot of experiences and perspectives during that period. Data collection began with an information session for HCWs and the distribution of an information sheet, with permission from the department head. A questionnaire with open- and closed-ended questions assessed HCWs’ perspectives on mental health screening and management for PLHIV, training background, availability of mental health providers, facility conduciveness, standard operating procedures (SOPs) and guidelines, comfort in managing mental disorders, and views on mental health prevalence among PLHIV. The Drop-off/Pick-up (DOPU) method was used to improve response rates.^18^ In-depth interviews employed a semi-structured guide to explore HCWs’ opinions on mental health service provision. Interviews were conducted privately at health facilities to ensure confidentiality and lasted on average 30 min. A recorder was used, and handwritten notes were taken by the principal investigator (PI). Concept saturation determined the number of interviews, with no follow-ups. The principal investigator and health assistants fluent in English conducted the IDIs, using probing as needed to clarify responses.

### Data analysis

Questionnaire data were captured in Microsoft Excel and analysed in STATA version 15 (College Station, TX, United States), with data preparation and cleaning. Descriptive statistics, including frequencies and proportions for categorical variables, were used to explore the data. Responses were summarised in tables, presenting means for continuous variables and proportions for categorical variables.

Qualitative data were transcribed in English, and translation was unnecessary. Transcribed scripts were coded using NVivo 12 (QSR International Pty Ltd, Victoria, Australia) using thematic analysis. We employed inductive methods to analyse the data. Key features of a general inductive approach include condensing large volumes of raw text data into a summarised form and establishing clear connections between research objectives and findings.^19^ An inductive approach involves identifying themes closely tied to the data; hence, inductive analysis entails coding data without attempting to fit it into an existing coding framework.^20^ The inductive analysis process followed Creswell’s 2002 five-step approach. The analysis followed several steps: data preparation and cleaning by formatting the raw data in Excel, followed by a close reading of the texts to identify emerging themes. Categories were then developed by identifying main themes aligned with the research aims and generating sub-themes through NVivo coding. Overlapping codes were reviewed and irrelevant texts excluded. Finally, themes and sub-themes were continuously refined during analysis and article preparation.^15^

### Trustworthiness of the study

All healthcare workers reported being fluent in English and were interviewed in English. The PI conducted all IDIs. The principal investigator has prior research expertise in the health sector and is knowledgeable about HIV service delivery in Namibia. Furthermore, the principal investigator has academic training and expertise in conducting qualitative interviews. Peer debriefing with a mentor was done to enhance reflexivity. To strengthen credibility, interviews were carefully transcribed through repeated reading of the transcripts and independent coding by two researchers, followed by regular discussions of the analysis. Transferability was supported by clear descriptions of the study setting and participants, enabling readers to assess whether the findings may apply to similar contexts. Dependability was supported through a systematic, transparent data analysis process, including the use of NVivo for coding and regular discussions between the researchers to refine themes. Confirmability was promoted through reflexive practices, including field notes and debriefing with a mentor to reflect on potential researcher bias.

### Ethical considerations

Ethical clearance to conduct this study was obtained from the University of KwaZulu-Natal Biomedical Research Ethics Committee (No. BREC/00002904/2021) and the Ministry of Health and Social Services’ ethical committee (No: 17/3/3/NKC). The study involved minimal risk, and respondents gave verbal consent to participate and have the interviews recorded. Participants were allowed to withdraw from the study at any time and skip any questions they were uncomfortable answering. No personally identifying information, such as first and last names or identity numbers, was collected to ensure participants’ anonymity. All data were transferred daily from the recorder to a password-protected computer and then deleted from the recorder. Only the principal investigator (PI) had access to the password-locked computer. Questionnaires were kept in a lockable drawer accessible only to the PI.

## Results

### Demographic characteristics of study participants

Table 1 outlines the demographic characteristics of the study participants. A total of 44 HCWs were included in the study, of which 34 were surveyed, and 10 participated in in-depth interviews. Most participants (43.2%) are nurses (*n* = 19/44), and half of the respondents (50%) reported having an educational qualification (*n* = 17/44) (Table 1). Most respondents work at a district hospital (36.4%, *n* = 16/44) and the PHC clinic (31.8%, *n* = 18). Additionally, most participants (32.4%, *n* = 11) indicate that they have been working at their respective health facilities for 1–4 years (Table 1).

**TABLE 1:** Demographic characteristics of survey respondents (*N* = 44).

Characteristics	ART site (*n* = 35)		Mental health site (*n* = 9)		Grand total	
	*n*	%	*n*	%	*n*	%
**Role of HCW (position)**						
Clinical mentor	2	6	-	0.0	2	4.5
Doctor	1	3	-	0.0	1	2.3
Health assistant	5	14	-	0.0	4	9.1
Medical intern	0	0	5	55.6	5	11.4
Medical officer/doctor	5	14	3	33.3	8	18.2
Nurse	19	54	-	0.0	19	43.2
Nurse mentor	3	9	-	0.0	3	6.8
Specialist	-	0	-	0.0	1	2.3
Total	35	100	9	100.0	44	100.0
**Highest education attained**						
Certificate	2	8	-	0.0	2	5.9
Degree	11	44	6	66.7	17	50.0
Diploma	4	16	-	0.0	4	11.8
Honours degree	3	12	-	0.0	3	8.8
Master’s degree	5	20	3	33.3	8	23.5
Missing	10	29	0	0.0	10	23.7
Total	25	71	9	-	34	77.0
**Type of health facility**						
Clinic	14	40	-	0.0	14	31.8
District hospital	7	20	9	100.0	16	36.4
Health centre	8	23	-	0.0	8	18.2
Intermediate hospital	1	3	-	0.0	1	2.3
National level	5	14	-	0.0	5	11.4
Total	35	100	9	100.0	44	100.0
**Duration of working at the hospital (years)**						
< 1	2	8	5	55.6	7	20.6
> 10	6	24	1	11.1	7	20.6
1–4	9	36	2	22.2	11	32.4
5–7	6	24	1	11.1	7	20.6
Unknown	2	8	0	0.0	2	4.5
Missing	10	28.5	0	0.0	10	22.7
Total	25	71.4	9	100.0	34	77.3

*Source*: Conteh, NK, Mahomed O. Data from the survey were put in tables for this manuscript. 2022. (unpublished)

ART, antiretroviral therapy; HCW, healthcare worker.

### Thematic analysis of results

The study findings were analysed using the Wakida et al. framework,^21^ which is adapted from the Supporting the Use of Research Evidence (SURE) and the Capability, Opportunity and Motivation-Behaviour (COM-B) frameworks. The SURE framework effectively identifies implementation factors, but does not offer concrete measures to resolve these issues.^21^

This study’s findings yielded common themes from the survey and key informant interviews. The main findings from the study are analysed and presented under four of the five SURE domains: (1) Motivation to change, (2) Knowledge and skills, (3) Management and leadership, and (4) Financial resources. Additionally, the findings are summarised and presented according to the SURE framework as well as the Wakida et al. framework of barriers.^21^

#### Domain: Motivation to change

**Barrier: Convenience of services:** Motivation to change is influenced by how convenient and supportive health facilities are for mental health service delivery. Health facility space and conduciveness were reported as barriers to service provision. Most respondents believe that health facilities are not conducive to mental health service provision. They reported that the lack of physical space and infrastructure at most health facilities is challenging, as many facilities lack adequate space to ensure a supportive mental health service environment, which compromises privacy. They emphasised the need for designated mental health areas:

‘… the spaces in the facility are limited, so maybe we need to find more space.’ (Health Assistant, 28 years)

#### Domain: Knowledge and skills

**Barrier: Inability to diagnose and treat mental illness and lack of knowledge regarding psychosocial interventions:** Effective integration of mental health services into HIV care is heavily influenced by the knowledge and skills of health workers, particularly their ability to identify, diagnose, and manage mental health conditions. Limited knowledge and skills in managing mental illness were identified as major barriers to providing mental health services for PLHIV. Health workers at ART sites reported that mental health education is rarely offered due to limited expertise, as illustrated by one respondent:

‘We don’t talk much about mental health in our health education; we mostly concentrate on the physical well-being of the client.’ (Health Assistant, 28 years)

Routine screening was inconsistent across facilities. Most ART providers indicated that PLHIV are not regularly screened for mental illness, while psychiatric outpatient staff reported limited HIV screening among patients with mental disorders. Some respondents noted that underreporting is common and that symptoms may go unnoticed without proactive assessment. As one participant stated:

‘I think it is under-reported because the patients that we usually refer are the ones with signs and symptoms, and those that are going through depression or something, but not showing signs, we really don’t pick them up if we don’t ask.’ (Doctor, 32 years)

Screening practices were largely informal, often triggered by concerning behaviour or reports from relatives. One nurse explained:

‘Sometimes the relatives bring the patient … saying he has been hearing and seeing things, then you start to suspect something is not right.’ (Nurse, 38 years)

Alcohol use was the only area with a formal tool, with general mental health screening conducted ad hoc:

‘For alcohol, we have a screening tool … but for general mental health screening, we just see as we talk to the person.’ (Nurse, 54 years)

Human resource limitations further constrained service delivery. Few ART facilities had mental health professionals, and most relied on referrals to the psychiatric hospital. The absence of onsite social workers also hindered linkage to care:

‘… It would be easier if social workers were on-site so they [*patients*] can be linked to care.’ (HCW, 54 years)

Most ART facilities lacked SOPs or guidelines for mental health, and some psychiatric units lacked ART guidelines. Health workers emphasised the need for training, standardised tools, and job aids to support integration. As one noted:

‘Health care workers should be trained on how to assess whether someone has mental illness … and after training, they should put the tools in place.’ (HCW, 41 years)

Another highlighted the need to develop a training curriculum to facilitate integration.

#### Domain: Management and leadership

**Barrier: No in-service training in mental healthcare:** Effective leadership and management are critical for the successful integration of mental health services into HIV care, particularly through supporting staff capacity building and ongoing professional development. Limited in-service training on mental health management was identified as a major barrier to providing mental health services. Although most respondents (66.7%) reported receiving basic mental health training during their academic studies, only 14% had received any in-service training in their current roles. As a result, mental health is seldom included in routine patient education, as one respondent noted:

‘We do not talk much about mental health in our health education; we mostly concentrate on the physical well-being of the client.’ (Health Assistant, 28 years)

Healthcare workers emphasised the need for treatment guidelines, job aids, and training to support integration efforts, with one manager stating:

‘I think we need to sensitise our leaders to understand the importance of integration. It’s very vital.’ (Health Manager, 34 years)

At the psychiatric hospital, 78% of respondents had been trained in HIV screening, diagnosis, and treatment, though some reported relying on referrals due to limited mental health diagnostic skills. As one nurse mentor explained:

‘I don’t have the proper training or knowledge of diagnosing a patient with mental illness, so we mostly refer.’ (Nurse Mentor, 35 years)

#### Financial resources

**Barrier: Cost of hiring and supporting staff:** Adequate financial resources are critical for strengthening the health workforce and ensuring the delivery of integrated mental health services in HIV care. Financial resources were identified as essential for hiring additional staff and supporting existing personnel in delivering mental health services. Participants emphasised that staff shortages and heavy workloads hinder effective screening and limit the feasibility of integrating mental health services into routine HIV care. As one clinician noted:

‘It would be time-consuming if we provide it [*mental health services*] to every patient, and we won’t manage to see all patients in the queue.’ (HCW [doctor], 32 years)

Another added that employing the necessary experts requires funding and time:

‘It can be the experts that need to be employed, which will cost money.’ (Health Assistant, 28 years)

Human resource constraints were also highlighted as a barrier, particularly the high patient-to–health worker ratio, which compromises the quality of screening. A manager described this challenge:

‘You might want to do a proper screening, but looking at the long queue, you might not do justice to this one client.’ (Health Manager, 35 years)

## Discussion

This study explored healthcare workers’ perspectives regarding barriers to integrating mental health services into ART services, using a framework adapted from Wakida et al.,^21^ which incorporates domains from the SURE and COM-B frameworks. Four primary barriers were identified: insufficient facility space, limited knowledge and skills to manage mental illness, lack of in-service mental health training, and constrained human resources.

Lack of space emerged as a major structural challenge affecting confidentiality and service provision. According to Wakida et al., the convenience of service provision is a key enabler for integration.^21^ Similar constraints have been reported in Liberia, where all 19 assessed facilities had limited space,^22^ and in Ethiopia and South Africa, where infrastructure limitations, including consulting rooms, equipment, and supplies, negatively affected integration sustainability.^23,24^ Although a Namibian study reported that 62% of respondents agreed that facility infrastructure met quality improvement standards,^25^ the present study suggests that space constraints may be localised. Evidence also shows that healthcare workers may perceive infrastructure as inadequate even when objective assessments indicate otherwise,^23^ highlighting the importance of Opportunity factors in the COM-B model. Addressing these barriers could include creating dedicated mental health spaces, reorganising existing infrastructure, and ensuring private consultation areas to support service integration.

Limited knowledge and skills, and the absence of in-service training, further hindered mental health service provision, reflecting a capability gap within the COM-B framework. Although many HCWs received basic training during their academic studies, the lack of ongoing in-service training led to significant knowledge gaps. This aligns with findings from Ethiopia, where HCWs reported insufficient skills to diagnose and manage mental illness.^26^ Respondents also attributed underreporting of mental illness to irregular screening and the absence of screening tools, consistent with studies highlighting inadequate training in evidence-based assessment tools as a barrier.^27,28^ The lack of SOPs and mental health guidelines further reduced HCWs’ confidence. This reflects broader national challenges, as mental health care in Namibia remains concentrated in two psychiatric hospitals. The availability of SOPs, guidelines, and continuous professional development would support standardisation, improve HCW competency, and enhance care quality.^29,30^

Limited human resources were also a key barrier. HCWs noted understaffing, heavy workloads, and insufficient financial resources to hire additional staff, consistent with global evidence showing a gap between mental health needs and workforce availability in low- and middle-income countries (LMICs).^31^ This reflects a motivation issue within the COM-B model, as leadership engagement and resource allocation are critical for enabling integration. Financial constraints limit recruitment due to competing health priorities and inadequate health budgets.^32,33^ Task shifting or sharing offers a feasible solution, having been successfully used to expand HIV testing, ART delivery,^34^ and Voluntary Medical Male Circumcision (VMMC) services in resource-limited settings.^35^ This approach could also support the integration of mental health services by redistributing tasks across different cadres.

These findings have several implications for policy and practice in Namibia. Integrating mental health services into PHC ART clinics will require investments in physical infrastructure and designated consultation spaces. National guidelines and SOPs for mental health screening and treatment should be developed and disseminated to support standardisation across facilities. Well-structured in-service training programmes and continuous professional development initiatives are critical to improving HCWs’ capabilities, while task-shifting strategies, leadership engagement, and the allocation of financial resources can enhance opportunity and motivation. Based on these insights, immediate actions should focus on providing in-service training, SOPs, job aids, and temporary private consultation spaces. In the medium term, permanent designated mental health areas, standardised screening tools, and supervision and mentoring should be established. Longer-term strategies should integrate mental health into national PHC and ART policies, allocate sustainable financial resources, and institutionalise task-shifting approaches. Collectively, these steps provide a practical, phased roadmap for the sustainable integration of mental health services into ART care in Namibia, addressing the critical domains of skills, resources, and leadership engagement.

### Limitations

This study has several limitations. Snowball sampling may have introduced selection bias, as participants were referred by colleagues who may share similar perspectives. The small qualitative sample limits generalisability, although in-depth interviews allowed for detailed exploration of healthcare workers’ experiences, and key themes reached saturation. Conducting the study only in urban ART facilities further restricts generalisability to rural or other settings in Namibia. Social desirability bias may have influenced participants’ responses, despite assurances of confidentiality and encouragement to share honest experiences. Additionally, as the principal investigator conducted the interviews, responses may have been influenced by the researcher’s presence; reflexive practices, including debriefing with a mentor, were employed to minimise potential bias. This study used qualitative data from surveys and interviews, with written responses potentially providing less depth than interviews or focus groups. Combining perspectives of doctors, nurses, and nurse mentors may have obscured subtle differences between their viewpoints. Additionally, the study did not explore attitudes related to programme acceptability, appropriateness, or credibility, warranting further research. Despite these limitations, the study offers valuable insights into healthcare workers’ perspectives on barriers to integrating mental health into ART services, which can inform policy and practice in Namibia.

## Conclusion

The study found key barriers to mental health service provision in PHC settings in Namibia, such as staff shortages, limited facility space, and a lack of guidelines, knowledge, and skills among healthcare workers. These issues hinder effective screening, diagnosis, and treatment of mental illness in PLHIV, obstructing integration into primary care. Solutions include reorganising facilities, training healthcare workers, implementing task-shifting, and securing political commitment to invest in infrastructure. Importantly, these findings highlight the need for national policy initiatives to integrate mental health into HIV care guidelines, allocate resources for workforce development, and standardise service delivery across PHC facilities in Namibia.
